# Differential expression of circRNAs of testes with high and low sperm motility in Yili geese

**DOI:** 10.3389/fgene.2022.970097

**Published:** 2022-09-26

**Authors:** Yingping Wu, Haiying Li, Xiaoyu Zhao, Gulnar Baki, Chen Ma, Yingying Yao, Jiahui Li, Yang Yao, Lin Wang

**Affiliations:** College of Animal Science, Xinjiang Agricultural University, Urumqi, China

**Keywords:** yili geese, sperm motility, testis, circrnas, transcriptomic

## Abstract

The aim of this study was to explore the potential biological function of circular RNAs (circRNAs) in the sperm motility traits of Xinjiang Yili geese, and to provide a reference for analyzing the mechanism of regulation of Yili geese sperm motility. The 10 selected Xinjiang Yili Geese with high or low sperm motility (five for each group) were 3 years old, in good health, and were kept in the same feeding conditions. Yili geese were slaughtered for the collection of testicular tissue and high-throughput sequencing technology was used to screen differentially expressed circRNAs for bioinformatics analysis. Combined with the previously screened miRNAs related to the sperm motility of Yili geese, the circRNAs miRNAs regulatory network was constructed. The results showed that a total of 26,311 circRNAs were obtained from testicular tissues with high and low sperm motility, and 173 DECs were screened between the two groups (*p* < 0.05, |log2Foldchange|>0), of which 82 were up-regulated and 91 were down-regulated. Functional analysis of the source genes of these DECs showed that the source genes were mainly involved in biological processes. KEGG enrichment analysis showed that the source genes of DECs were mainly enriched in autophagy-animal, ubiquinone and other terpenoid-quinone biosynthesis, progesterone-mediated oocyte maturation, regulation of the actin cytoskeleton and other pathways. Furthermore, the visual regulatory network of differential circRNA-miRNA-mRNA was constructed, including 20 circRNAs, 18 miRNAs and 177 mRNAs, and nine core regulatory circRNAs were screened, including novell_circ_0045314, novel_circ_0019994 and novel_circ_0020422, etc., targeting ppy-mir-16, hsa-mir-221–3p, gga-mir-499–5p, etc. The results suggest that circRNAs may interact with miRNAs to further regulate mRNA to regulate sperm motility in Yili geese, so as to provide a reference for analyzing the molecular mechanism of sperm motility regulation.

## 1 Introduction

The Yili goose is the only local poultry variety in China that is derived from the herbivorous characteristics of gray geese. As a high-quality local characteristic poultry, the Yili goose has the characteristics of strong adaptability, good flight, heat resistance, cold resistance, rough feeding resistance, strong disease resistance and stress resistance, as well as good meat quality (Zhao et al., 2020). Reproductive traits are the most important economic traits in poultry production. Improving reproductive performance is always an important goal of poultry genetic improvement. The semen quality of male birds is directly related to the fertilization ability of sperm, which is one of the important indicators affecting reproductive efficiency. According to previous research, the low fertility of male Yili geese and the low fertilization rate of eggs limit the development of the Yili goose industry (Zhao et al., 2019). We also found that the sperm motility of yili geese was significantly different among individuals, and there was a positive correlation between sperm motility and fertilization rate (Wu et al., 2022). Therefore, there is an urgent need to use molecular biotechnology to improve the reproductive performance of male Yili geese and to explore the genetic mechanisms affecting their semen quality.

CircRNAs are closed circular RNA transcripts formed by reverse splicing from a single RNA precursor and are found in all higher eukaryotes, including mammals (Li et al., 2018). It has been found that circRNA plays an important role in organisms. It can be used as competitive endogenous RNA (CeRNA) or as an “miRNA sponge” to inhibit the activity of miRNA, weaken or even relieve the inhibitory effect of miRNA on downstream target genes, and promote the expression of target genes (Zhong et al., 2018). At present, there are many studies on the roles of circRNAs in various aspects of the mammalian reproductive system, such as testicular development (Li et al., 2021; Zhang et al., 2021), follicle development (Guo et al., 2020; Liang et al., 2020), embryonic development (Quan and Li, 2018), etc. However, studies on the molecular mechanisms of circRNAs in the poultry reproductive system have mainly focused on the follicle development of female poultry (Shen M. et al., 2019; Shen M. et al., 2020; Wu et al., 2020). Research on the mechanism of regulation of circRNA with regard to sperm motility in the Yili goose is still lacking. In this study, Yili geese with extreme differences in sperm motility were taken as the objects of research. The expression pattern of circRNA in the testicular tissue of Yili geese with high and low sperm motility was analyzed by RNA-seq technology, and the circRNA related to the sperm motility of male geese was screened and identified, so as to provide a theoretical basis for the genetic and breeding improvement of Yili geese.

## 2 Materials and methods

### 2.1 Sample collection

The experimental animals used in this study were provided by Hengxin Industrial Co., Ltd. Emin County, Xinjiang. According to the data on sperm motility and egg fertilization rate, among the 3-year-old Xinjiang Yili goose with similar body weight (3.65±0.40 kg) and the same feeding conditions, 5 geese with high and low sperm motility were selected (*p* < 0.01) (Table 1). After the geese were sacrificed, their testis tissues were collected immediately, and stored in liquid nitrogen, rinsed with PBS 1 to 2 times, and immediately placed in a cryopreservation tube containing RNA preservation solution. The testis samples of the HFR geese (high sperm motility group) were labeled as HFR-1∼HFR-5, and the testis samples of the LFR geese (low sperm motility group) were labeled as LFR-1∼LFR-5. The samples were labeled and stored at 4 °C overnight and then stored at -80 °C the next day until they were used for the extraction of total RNA.

**TABLE 1:** Sperm motility and fertilization rate of Yili geese with high and low sperm motility.

Item	HFR-1 (%)	HFR-2 (%)	HFR-3 (%)	HFR-4 (%)	HFR-5 (%)	Mean ± SD	LFR-1 (%)	LFR-2 (%)	LFR-3 (%)	LFR-4 (%)	LFR-5 (%)	Mean ± SD
Sperm motility (%)	77.31	70.73	63.27	58.40	58.42	65.63%±8.25%	31.67	37.48	32.46	39.49	38.09	35.84%±3.53%
Fertilization rate (%)	98.33	97.50	94.17	93.24	91.77	95.00%±2.81%	40.99	43.45	43.50	49.36	46.67	44.79%±3.25%

### 2.2 Total RNA extraction and illumina sequencing

The total RNA was extracted using Trizol (Invitrogen, Carlsbad, CA, United States), following the manufacturer’s protocol, and agarose gels, a Nanodrop ND-1000 spectrophotometer (IMPLEN,CA, United States) and an Agilent 2100 Bioanalyzer (Agilent Technologies, CA, United States) were used to ensure that the quality of the samples was sufficient for transcriptome sequencing. After the samples passed quality control, the strand-specific library was constructed by removing ribosomal RNA (circRNA library building and the linear RNA process). After the libraries were qualified, the library preparations were sequenced on an Illumina Hiseq platform and 150 bp paired-end reads were generated.

### 2.3 circRNAs identification

Raw data (raw reads) in fastq format were firstly processed through in-house perl scripts. In this step, clean data (clean reads) were obtained by removing reads containing adapter, reads containing ploy-N and low-quality reads from raw data. At the same time, for Q20, Q30 and GC content the clean data were calculated. The index of the reference genome (Yan et al., 2020) was built using Bowtie2 (v2.2.8) (Langmead et al., 2009)and paired-end clean reads were aligned to the reference genome using Bowtie. The circRNA was detected and identified using find_circ (v1.2) (Memczak et al., 2013)and CIRI2 (v2.0.5) (Gao et al., 2015), and the intersection of the two types of software was also used to identify the circRNA.

### 2.4 circRNAs difference analysis and functional enrichment analysis

HTSeq v0.6.0 (Anders et al., 2015)was used to count the reads numbers mapped to each gene, and the expression amount was normalized with TPM (Zhou et al., 2010). The input data of circRNA differential expression is readcounts data obtained from circrna expression level analysis. Differential expression analysis of the two groups was performed using the DESeq2 R package (1.10.1) (Michael et al., 2014). circRNAs with a *p* < 0.05 and |log2Foldchange|>0 found by DESeq2 were set as the threshold for significantly differential expression. The GOseq (Release 2.1.2) (Young et al., 2010) software was used for the GO enrichment analysis of differentially expressed circRNA-derived genes, and KOBAS (2.0) (Mao et al., 2005; Kanehisa et al., 2008) was used for Paththe way enrichment analysis. A *P* of <0.05 was set as the threshold for statistically significant results.

miRNA target sites in the exons of circRNA loci were identified using miRanda (Enright et al., 2003). miRNA target genes were predicted to be the intersection of miRanda (Enright et al., 2003) and RNAhybrid (Kruger and Rehmsmeier, 2006). Cytoscape (v3.7.1) (Paul et al., 2003) software was used to construct the circRNA-miRNA-mRNA networks.

### 2.5 Real-time PCR validation of sequencing results

Randomly selected from the transcriptome sequencing results, 8 DECs related to the HFR and LFR of Xinjiang Yili geese were used for fluorescence-based quantitative validation. Oligo 7.0 software was used to design primers (Supplementary Table S1). SYBR GREEN reagent (TaKaRa) was used to amplify the target gene and internal reference gene (beta-actin) mRNAs on a ROCHE 480 quantitative PCR instrument (Eppendorf, Germany). The PCR reaction system (20 μl) included the 10 μl AceQ Universal SYBR qPCR Master Mix, 0.4 μl upstream primer (10 μmol L/L), 0.4 μl downstream primer (10 μmol L/L), 6.7 μl ddH_2_O, and 2.5 μl cDNA. The reaction conditions were as follows: 95°C for 5 min, 95°C for 10 s, 60°C for 30 s, 40 cycles; dissolution curve: 60°C→95°C, with a temperature increase of 0.3°C every 15 s. Quantitative expression results were calculated according to the cross point (CP) values, and the relative expression levels were calculated according to the 2^−∆∆Ct^ method (Bustin et al., 2009).

## 3 Results

### 3.1 Screening of yili geese with high and low sperm motility

Based on the semen quality data from six instances and the fertilization rate data from five instances, five individuals in the high sperm motility group and five individuals in the low sperm motility group were screened out. An independent t-test showed that the sperm motility and fertilization rate of the individuals in the high sperm motility group were significantly higher than in the low sperm motility group (*p* < 0.01, Table 1).

### 3.2 Evaluation of sequencing data quality

It can be seen from Table 2 that 495,401,294 clean reads and 475,402,620 reads were obtained by sequencing Yili geese with high and low sperm motility, respectively. The Q20 and Q30 of each sample were at least 97.49% and 93.06%, respectively, and the GC contents were between 45.40% and 47.40%. These results indicated that the quality of transcriptome sequencing results met the needs of subsequent analyses. According to the statistical results, 91.29%–92.37% of the clean reads were mapped to the reference genome, of which 86.06%–89.40% of the clean reads were uniquely mapped. The results showed that there were enough reads of each sample mapped to the reference genome, and the selected reference genome was suitable.

**TABLE 2:** Evaluation of sequencing data quality.

Sample name	Raw reads	Clean reads	Q20 (%)	Q30 (%)	GC content (%)	Total mapped	Uniquely mapped
HFR_1	86,345,158	85,695,512	97.65	93.43	47.19	78,316,871 (91.39%)	73,750,086 (86.06%)
HFR_2	105,769,898	104,987,610	97.63	93.38	46.50	96,332,847 (91.76%)	92,098,738 (87.72%)
HFR_3	93,937,816	93,265,686	97.49	93.06	46.53	85,686,995 (91.87%)	82,404,051 (88.35%)
HFR_4	105,232,800	104,604,358	97.63	93.39	46.81	96,203,761 (91.97%)	92,639,975 (88.56%)
HFR_5	107,655,208	106,848,128	97.61	93.36	47.22	98,144,265 (91.85%)	94,536,194 (88.48%)
LFR_1	104,869,524	103,850,998	97.60	93.37	47.40	95,013,805 (91.49%)	90,797,347 (87.43%)
LFR_2	82,214,726	81,594,444	97.68	93.48	46.60	74,489,752 (91.29%)	71,049,348 (87.08%)
LFR_3	106,378,240	105,732,972	97.60	93.28	45.40	97,160,534 (91.89%)	94,049,495 (88.95%)
LFR_4	84,278,058	83,586,102	97.53	93.11	45.80	77,212,079 (92.37%)	74,728,549 (89.40%)
LFR_5	101,369,790	100,638,104	97.60	93.28	46.65	92,182,939 (91.60%)	87,737,183 (87.18%)

### 3.3 circRNAs identification and characteristic analysis

#### 3.3.1 Identification of circRNAs

**FIGURE 1:**
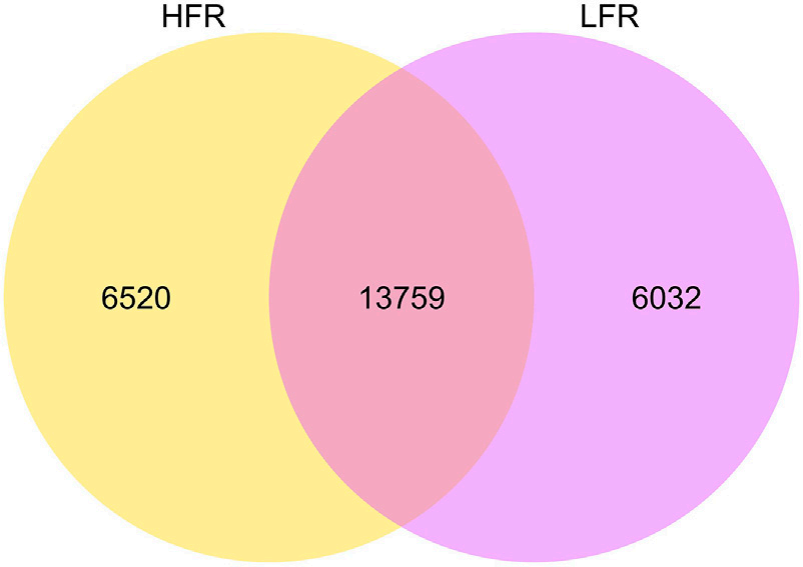
circRNAs difference between HFR and LFR. The big circle represents each comparison combination, the sum of the numbers in each big circle represents the total number of circrnas identified by the comparison combination, and the overlapping part of the circle represents the number of circrnas common between the combinations.

A total of 26,311 circRNAs were identified in testicular tissues (Figure 1), of which 6,520 and 6,032 were unique of the Xinjiang Yili geese with HFR and LFR, respectively. According to the annotation of the reference genome, it was found that many of the host genes of circRNAs are related to male reproduction (Table 3, Supplementary Table S2), such as spermatogenesis-related *SPA5L*, *SPAT5*, *SPAT6*, *SPT17*, *SPT48*, etc., testis-specific genes *TESK1*, *TEX10*, *TSG10* etc., and flagellar motility genes *SPEF2*, *CF206*, *IF172*, etc., showing that it is feasible to sequence testicular tissue by RNA-seq technology and to screen for biomarkers related to geese reproduction.

**TABLE 3:** Annotation of host genes associated with gander reproduction for partial circRNAs.

circRNAs	Host genes	Description	position
novel_circ_0013754	*SPA5L*	BOVIN Spermatogenesis-associated protein 5-like protein 1	chr19:22147438–22148362
novel_circ_0008443	*SPAT5*	MOUSE Spermatogenesis-associated protein 5	chr15:12275327–12294121
novel_circ_0007364	*SPAT6*	HUMAN Spermatogenesis-associated protein 6	chr14:23958879–23965428
novel_circ_0000297	*SPT17*	HUMAN Spermatogenesis-associated protein 17	chr10:17374912–17389052
novel_circ_0041302	*SPT48*	HUMAN Spermatogenesis-associated protein 48	chr8:26482947–26486091
novel_circ_0004385	*TESK1*	HUMAN Dual specificity testis-specific protein kinase 1	chr12:25506529–25508863
novel_circ_0041026	*TEX10*	CHICK Testis-expressed protein 10 homolog	chr8:19326415–19339250
novel_circ_0033683	*TSG10*	HUMAN Testis-specific gene 10 protein	chr4:37691305–37693198
novel_circ_0004352	*SPEF2*	RAT Sperm flagellar protein 2	chr12:23705501–23734451
novel_circ_0014569	*CF206*	MACFA Cilia- and flagella-associated protein 206	chr1:20634161–20635541
novel_circ_0014350	*IF172*	MOUSE Intraflagellar transport protein 172 homolog	chr1:141083–142558

#### 3.3.2 Characteristic analysis of circRNAs

**FIGURE 2:**
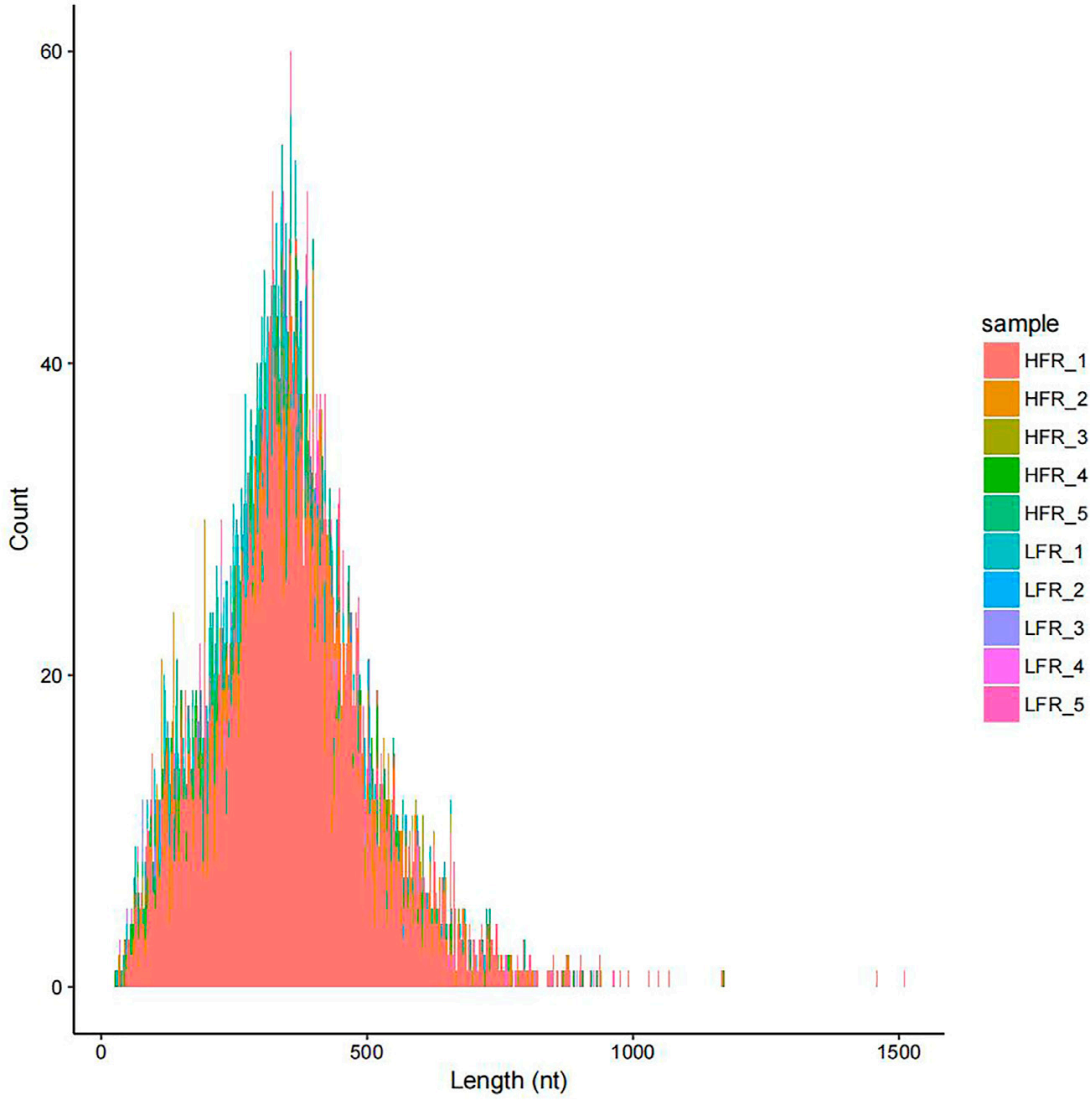
Length distribution of circRNAs.

**FIGURE 3:**
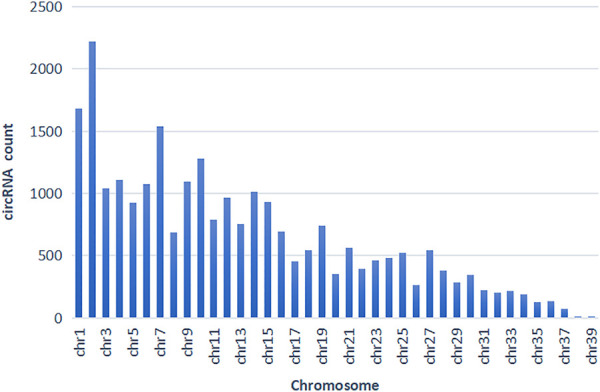
circRNA chromosome distribution.

**FIGURE 4:**
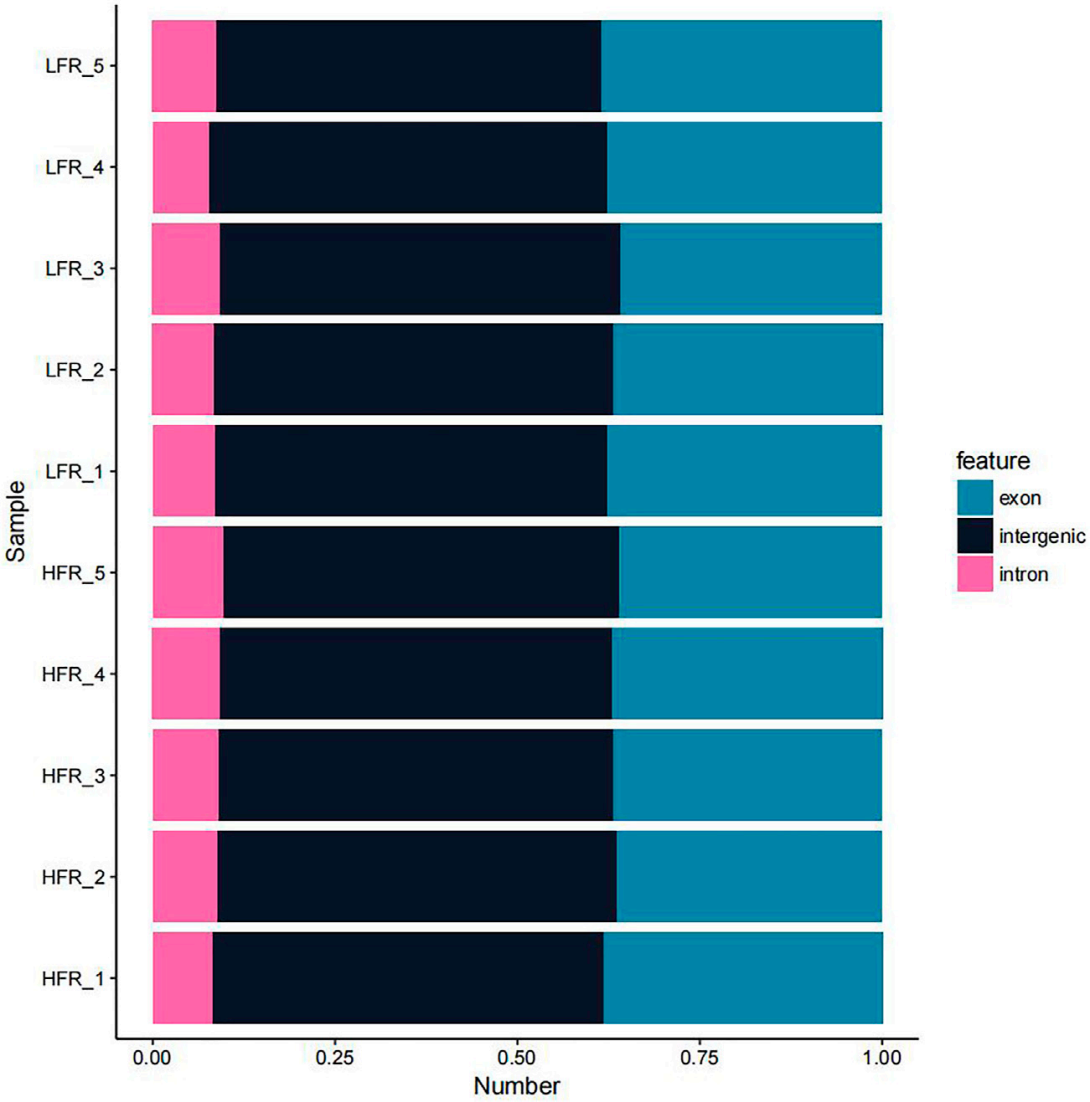
circRNA source statistics.

According to the comparison results of circRNAs and the reference genome, there were 14,232 circRNAs without corresponding host genes, and other circRNAs were mapped to 12,080 genes. The lengths of these circRNAs ranged from 23 bp to 1,511 bp, with an average length of 357 bp (Figure 2). Most circRNAs were distributed on chromosome 2, accounting for 8.43%, followed by chromosomes 1, 7, and 10, accounting for 6.38%, 5.83%, and 4.87%, respectively (Figure 3). According to their location in the genome, most circRNAs were derived from intergenic regions (34.19%), followed by exonic regions (33.45%) and intronic regions (32.36%) (Figure 4).

### 3.4 circRNAs differential expression analysis

**FIGURE 5:**
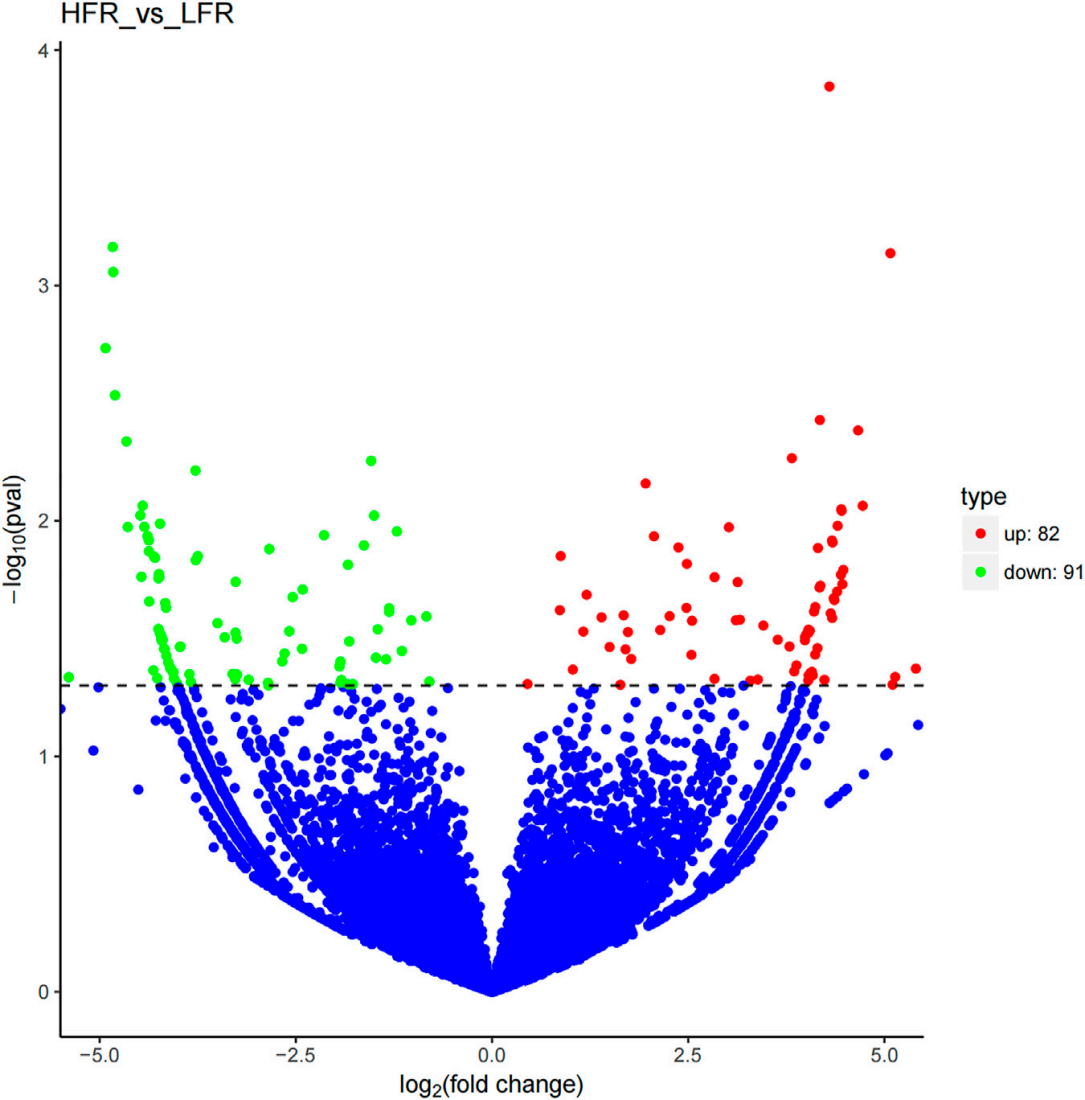
Differential circRNA volcano map. Differentially expressed circRNAs were filtered using a *p* < 0.05 as a threshold. Red spots represent up-regulated circRNAs, and green spots indicate down-regulated circRNAs. Blue spots represent circRNAs that did not show obvious changes between the HFR and LFR samples.

**FIGURE 6:**
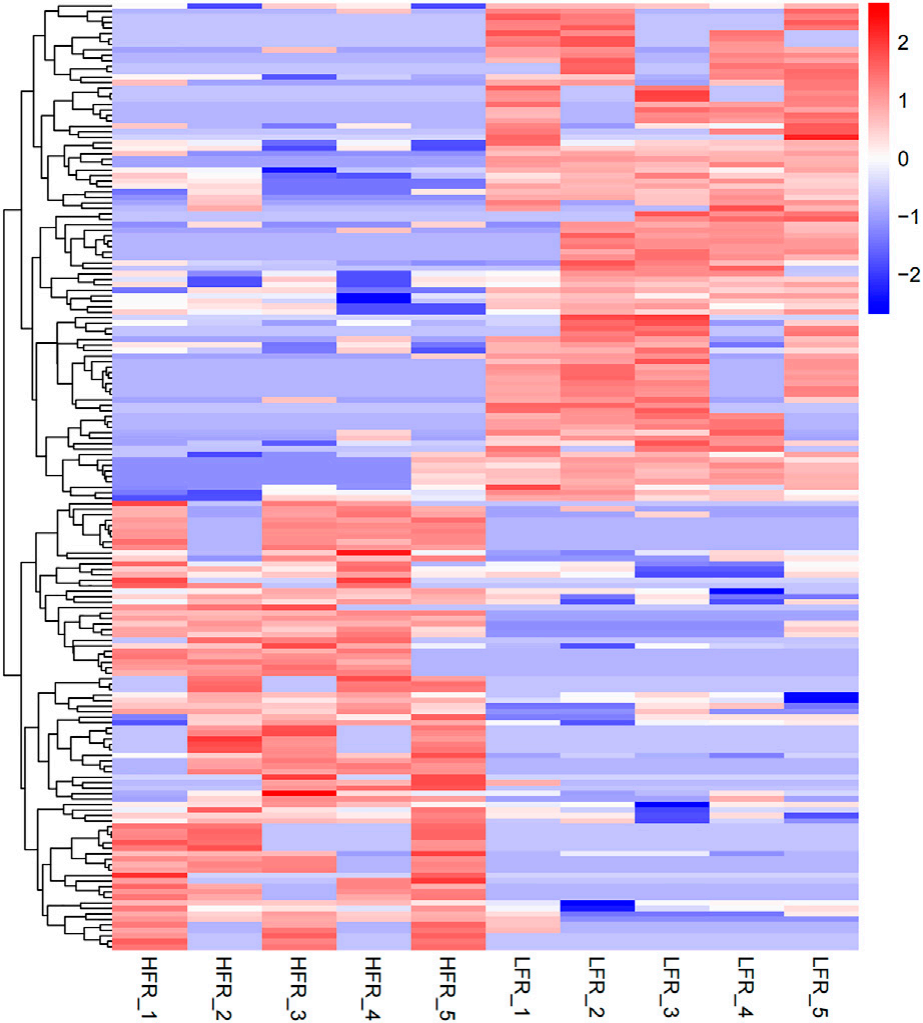
Differential circRNA cluster map. The horizontal axis is the sample, and the vertical axis is the differential gene. The left side clusters the genes according to the expression similarity, and the upper part clusters each sample according to the expression profile similarity. The expression level gradually increases from blue to red, and the number is the relative expression level after homogenization.

Compared with the low sperm motility group, a total of 173 DECs (differentially expressed circRNAs) were screened in the high sperm motility group, of which 82 were up-regulated and 91 were down-regulated (*p* < 0.05, |log2Foldchange|>0) (Figure 5), and 77 circRNAs had the source genes. Among them, the circRNAs with the smallest *p* values were novel_circ_0013769 and novel_circ_0007998, both located in the intergenic region, and both being up-regulated circRNAs. The fold difference was the largest in novel_circ_0042868 (source gene *PDE6C*), novel_circ_0018689 (intergenic_region) and novel_circ_0030568 (source gene *ADAM9*), with a fold difference of 7.6687, 7.1261 and 7.0849, respectively. There were 87 circRNAs with more than a 4-fold difference, accounting for 50.29% of the DECs. Hierarchical clustering analysis was performed on the circRNAs that were significantly differentially expressed in the testes of the two groups, as shown in the heat map (Figure 6), red color and blue color respectively represent the significant increase or decrease in the HFR group compared with the LFR group. It can be seen that the repeatability within the sample group was good, and the difference between the groups was large, which can better reflect the differences between different treatments.

### 3.5 Functional analysis of circRNA host genes

**FIGURE 7:**
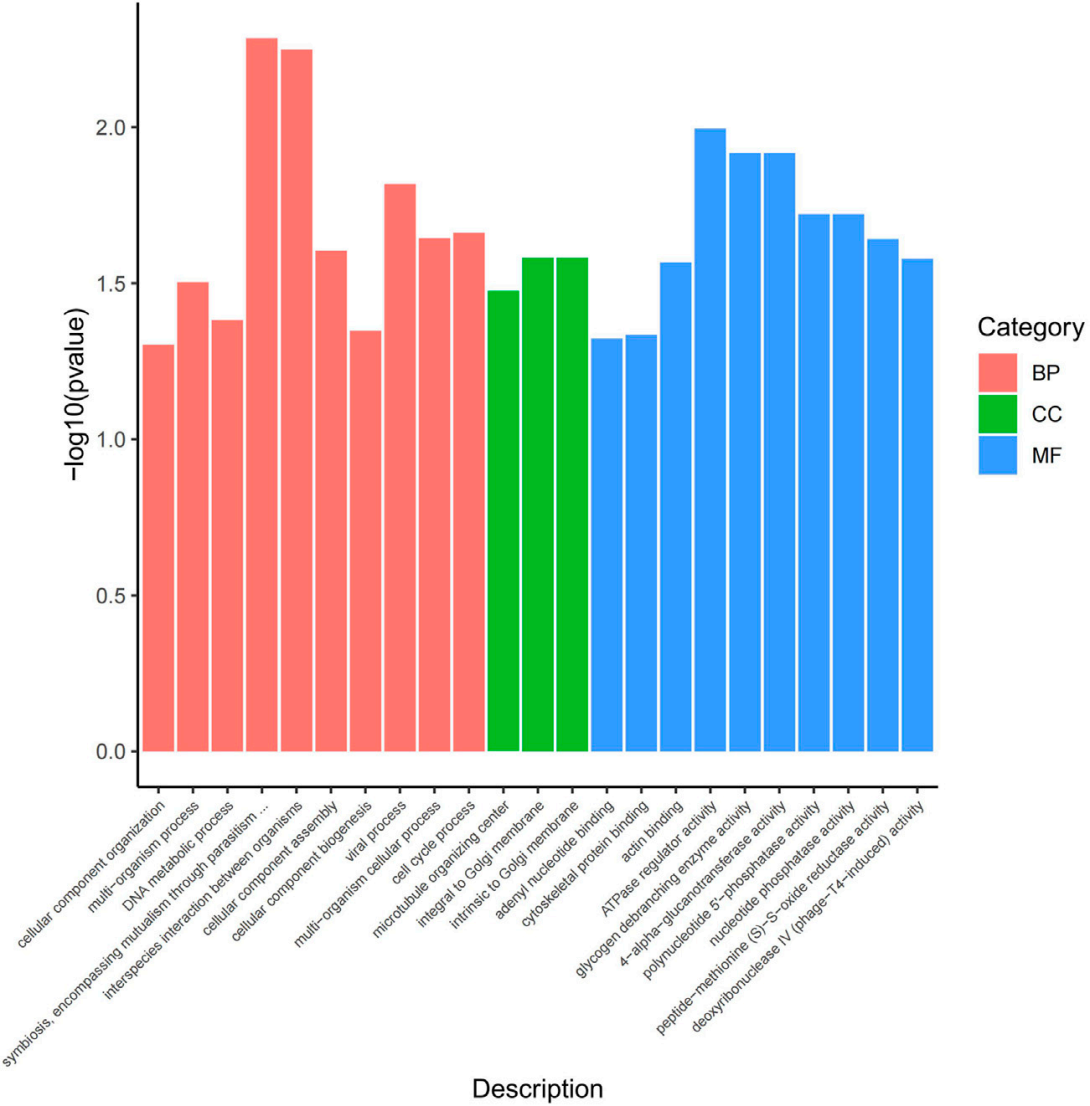
GO enrichment map. The horizontal axis represents the GO term of the next level of the three categories of GO, and the vertical axis represents the *p* value ranges.

**FIGURE 8:**
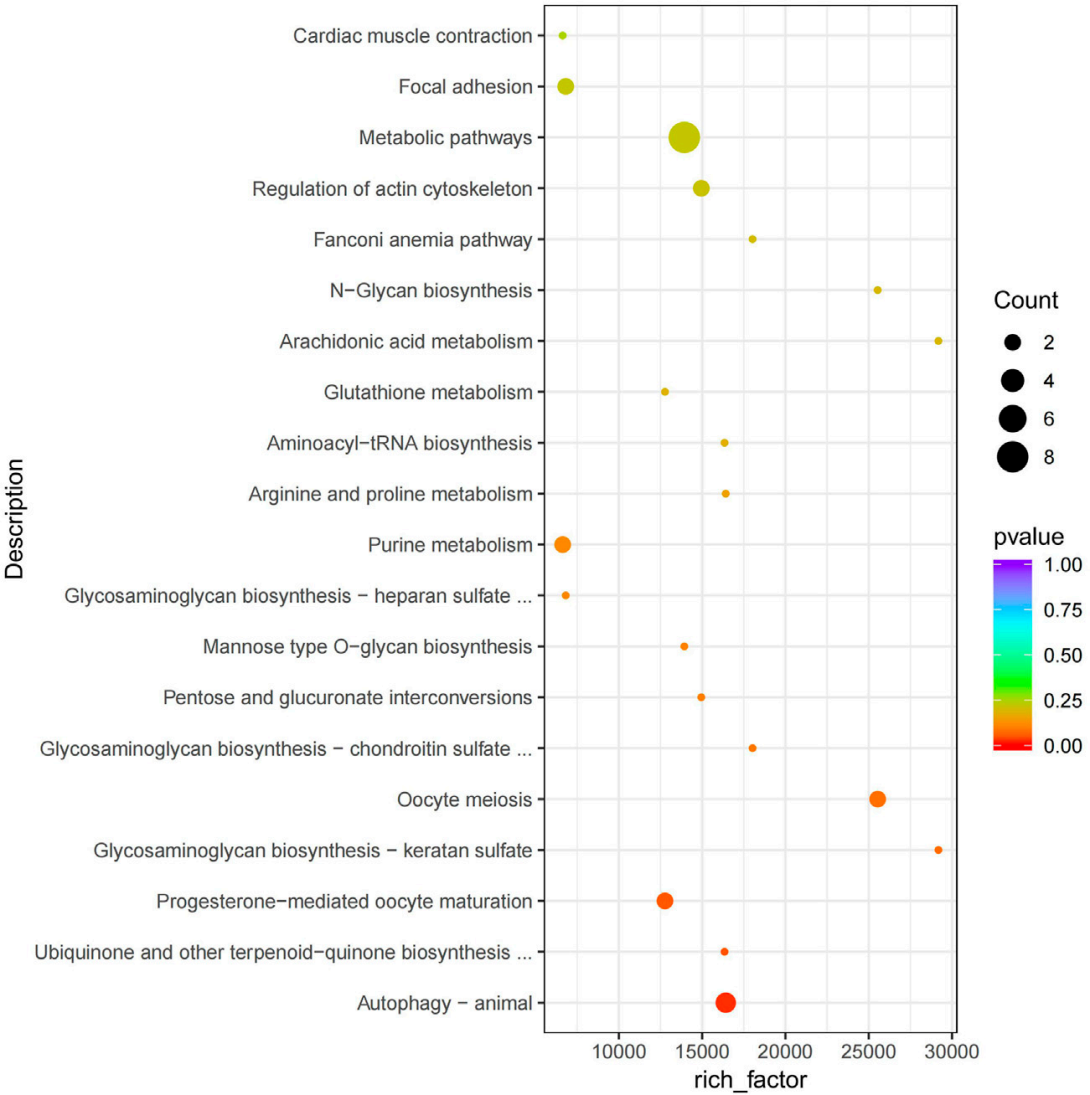
KEGG enrichment map. The vertical axis represents the pathway name, the horizontal axis represents rich factor, the size of the point represents the number of differentially expressed genes in this pathway, and the color of the point corresponds to different *p* value ranges.

The functional analysis of the source genes of these DECs (Figure 7; Table 4) showed that 58 significant GO terms were enriched (*p* < 0.05), and biological processes (63.79%) were mainly enriched in cellular component organization, multi-organism processes, and the reproduction of a single-celled organism. The molecular function (31.03%) was mainly enriched in adenyl nucleotide binding, cytoskeletal protein binding, actin binding, etc., and the cellular component (5.17%) was mainly enriched in the microtubule organizing center, integral to the Golgi membrane. KEGG analysis was mainly enriched in autophagy-animal, ubiquinone and other terpenoid-quinone biosynthesis, progesterone-mediated oocyte maturation, oocyte meiosis and purine metabolism and other signaling pathways, of which metabolic pathways were enriched. The pathways with the most genes, autophagy-animal and ubiquinone and other terpenoid-quinone biosynthesis were the most significantly enriched pathways, and the source genes of differential circRNAs involved in these two pathways were *Pdpk1* (phosphoinositide-dependent protein kinase 1) and *COQ5* (methoxy-6-polypropylene-1,4-benzoquinone methylase) (Figure 8;Table 5).

**TABLE 4:** GO enrichment analysis of circRNA host genes.

Item	GO Terms	*P*	Gene number	Gene names	GO_accession
Cellular component	microtubule organizing center	0.033481	2	*GULP1*,*ANGPT2*	GO:0005815
	integral to Golgi membrane	0.026197	1	*SIPA1L1*	GO:0030173
	intrinsic to Golgi membrane	0.026197	1	*SIPA1L1*	GO:0031228
Molecular function	adenyl nucleotide binding	0.047514	12	*RARS2*,*PRKCD*,*USP32*,A*rl15*,*NSF*,*KIF14*,*EXOC3*,*TTBK2*,*Pdpk1*,*RAB8B*,*BUB1*,*GULP1*	GO:0030554
	cytoskeletal protein binding	0.046215	5	*MAP7*,*Pdpk1*,*KIF14*,*ESPN*,*ANGPT2*	GO:0008092
	actin binding	0.027103	4	*Pdpk1*,*MAP7*,*ESPN*,*ANGPT2*	GO:0003779
	ATPase regulator activity	0.010083	2	*GULP1*,*STARD13*	GO:0060590
Biological process	cellular component organization	0.049637	9	*GULP1*,*SIPA1L1*,*MAP7*,*Pdpk1*,*EXOC3*,*HSF3*,*ANGPT2*,*CFAP43*,*DTNB*	GO:0016043
	multi-organism process	0.031369	7	*STARD13*,*GULP1*,*FUT8*,*HSF3*,*PLAC9*,*MAP7*,*NSF*	GO:0051704
	DNA metabolic process	0.04148	7	*NSF*,*ADAM9*,*EXOC3*,*GULP1*,*SYDE2*,*Dmd*,*STARD13*	GO:0006259
	symbiosis, encompassing mutualism through parasitism	0.0051803	6	*HSF3*,*FUT8*,*STARD13*,*GULP1*,*NSF*,*MAP7*	GO:0044403
	interspecies interaction between organisms	0.0056309	6	*FUT8*,*HSF3*,*STARD13*,*GULP1*,*NSF*,*MAP7*	GO:0044419

**TABLE 5:** KEGG enrichment analysis of circRNA host genes.

Item	KEGG pathway	*P*	Gene number	Gene names	KEGG_ID
KEGG PATHWAY	Autophagy-animal	0.017301176	3	*PRKCD*,*RB1CC1*,*Pdpk1*	acyg04140
	Ubiquinone and other terpenoid-quinone biosynthesis	0.046647725	1	*COQ5*	acyg00130
	Progesterone-mediated oocyte maturation	0.051907408	2	*Cpeb3*,*BUB1*	acyg04914
	Oocyte meiosis	0.079218003	2	Cpeb3,BUB1	acyg04114
	Metabolic pathways	0.223388493	8	*GMPR*,*ODC1*,*XYLT1*,*ALOX5*,*FUT8*,*COQ5*,*ispd*,*PDE6C*	acyg01100

### 3.6 Construction and analysis of circRNA-miRNA-mRNA interaction network

**FIGURE 9:**
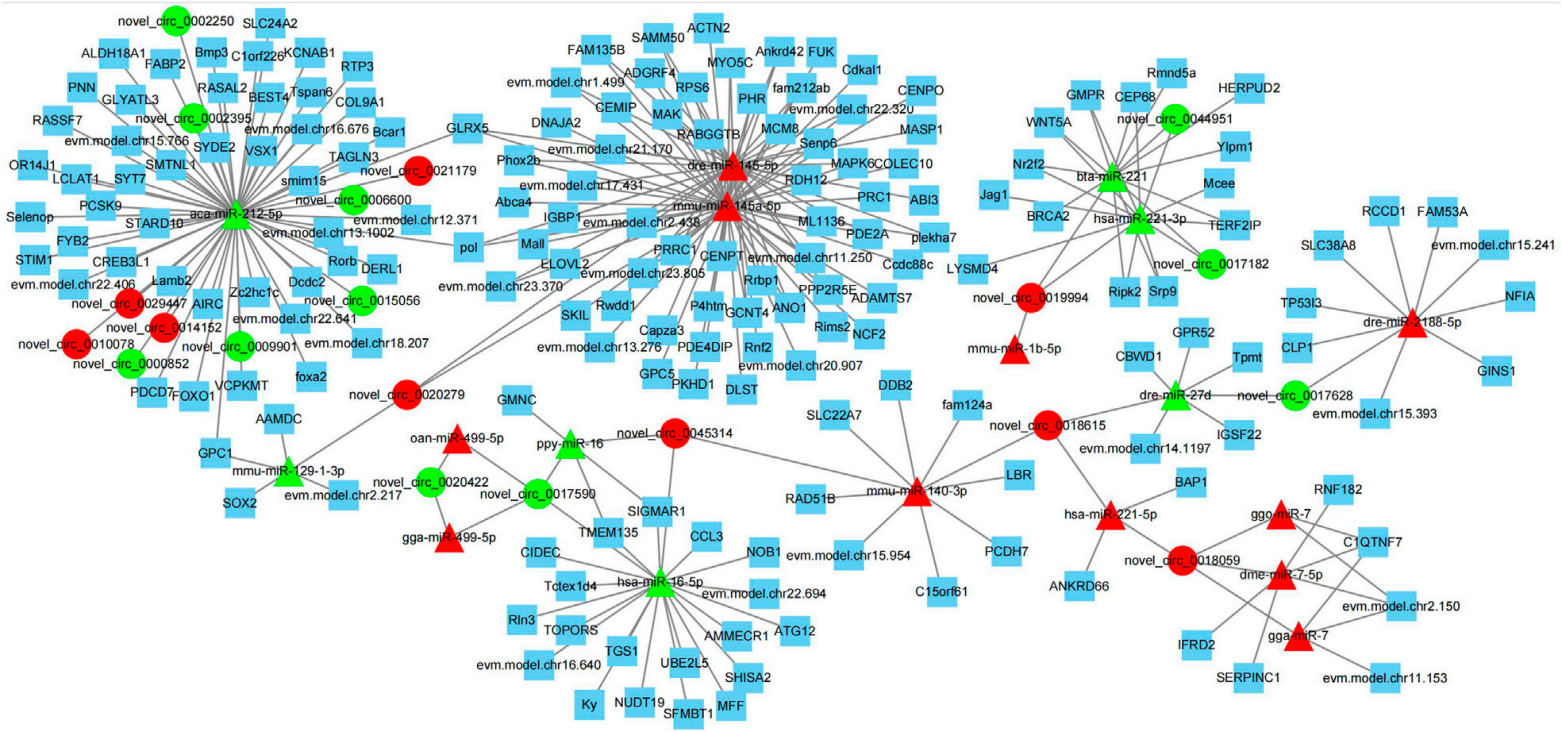
Interaction diagram of differential circRNA-miRNA-mRNA network. Triangle nodes represent the targeted miRNAs, circular nodes show circRNAs, and squares nodes show mRNA. Red color represent up-regulated, and green color indicate down-regulated.

circRNAs can adsorb miRNAs by combining with them, and can act as miRNA sponges (Li et al., 2018). Therefore, miRNA binding site analysis on the identified circRNAs is helpful to further study the function of circRNAs. In this study, the differentially expressed miRNAs in the testis of Yili geese with high and low sperm motility obtained in the previous stage were combined to construct a differential circRNA-miRNA-mRNA interaction network. As shown in Figure 9, the network was enriched with 20 circRNAs, 18 miRNAs and 177 mRNAs, and each circRNAs had at least 2 or more miRNA binding sites. Aca-mir-212–5p has 10 target circRNAs and 49 target mRNAs. Hsa-mir-221–3p has 3 target circRNAs and 10 target mRNAs. Bta-mir-221 has 3 target circRNAs and 11 target mRNAs. In addition, novel_ circ_ 0017590, novel_ circ_0018059 had four miRNA binding sites.

novel_ circ_ 0045314, novel_ circ_ 0019994 and novel_ circ_ 0020422 has two negatively regulated target miRNAs, novell_ circ_0017628, novel_ circ_ 0018615, novel_ circ_ 0021179, novel_circ_ 0029447, novel_ circ_ 0014152 and novel_ circ_ 0010078 has one negatively regulated target miRNA. These results suggested that circRNAs in testis may regulate testicular development and sperm motility by actively participating in binding with miRNAs, acting as competitive endogenous RNAs, regulating the function of target miRNAs, and thus indirectly targeting mRNA levels.

### 3.7 Fluorescence quantitative polymerase chain reaction

**FIGURE 10:**
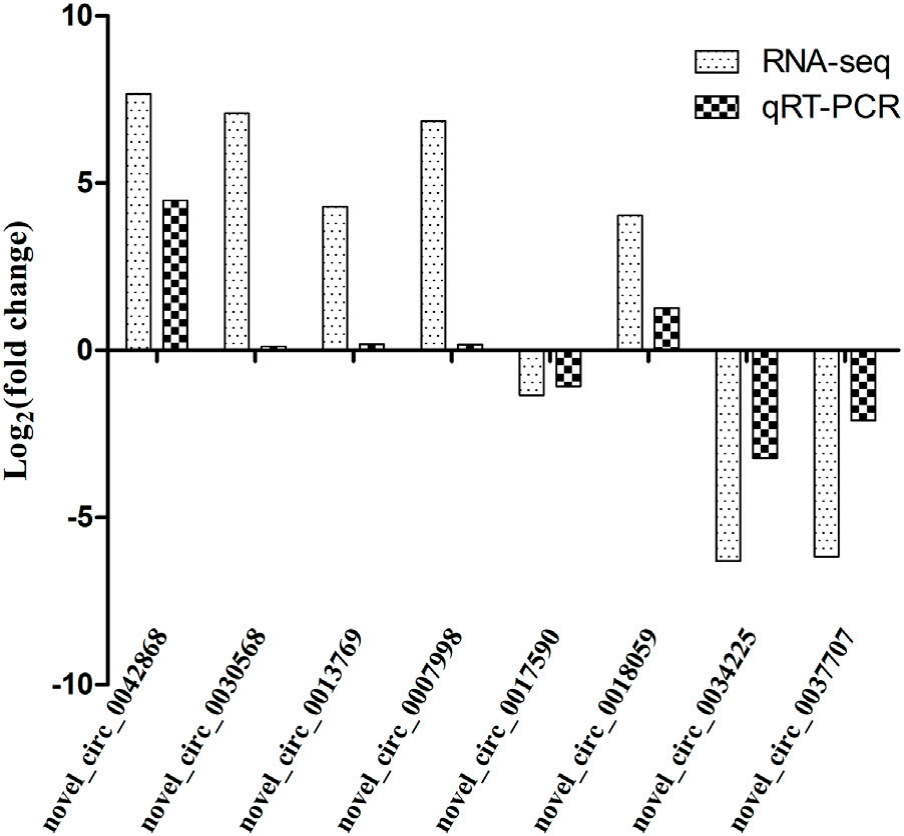
Comparison of qRT-PCR and RNA-seq results. Total RNA extracted from the hypothalami, pituitary glands, and ovaries tissues that were measured by qRT-PCR analysis; relative expression levels were calculated according to the 2^−∆∆Ct^ method using *GAPDH* as an internal reference gene.

In order to validate the differentially expressed genes identified by transcriptome sequencing, eight circRNAs were randomly selected and confirmed by qRT-PCR using *GAPDH* as the internal reference gene. The results showed that the expression trends of the eight circRNAs were consistent with those of the transcriptome sequencing results. Therefore, the transcriptome sequencing results are reliable and can be further studied and analyzed (Figure 10).

## 4 Discussion

Semen quality is one of the important indicators to measure the breeding value of male birds. The evaluation indicators of semen quality mainly include semen color, sperm motility, sperm viability, sperm deformity rate, semen volume, pH, etc (Jerysz and Lukaszewicz, 2013; Lukaszewicz et al., 2021). Sperm motility is a reflection of metabolic capacity, and is an important factor in ensuring that the sperm and egg meet and complete fertilization. Studies have shown that sperm motility traits are highly positively correlated with fertilization rate (Nguyen, 2019). Moreover, the evaluation of sperm motility traits is relatively simple compared with other semen quality traits, and is easy to generalize. Therefore, sperm motility is the most suitable indicator to measure the reproductive performance of breeding poultry. The testis is an important reproductive organ of male birds, and its main functions are spermatogenesis and endocrine function (Iddrriss et al., 2018). The seminiferous tubules are distributed inside the testis and are the main location of spermatogenesis, making them the most suitable organ for determining male sperm motility.

circRNAs are a new class of RNAs that are different from traditional linear RNAs. They are conserved in different species and are specifically expressed in tissues at different developmental stages. circRNAs can be regulated by affecting the transcription, mRNA conversion and translation of RNA-binding proteins and miRNAs(Panda, 2018; Kristensen et al., 2019). So far, research on circRNAs has mainly focused on humans and model animals, and less research has been carried out on testicular development. Like other vertebrates, the avian testis is the site of spermatogenesis and androgen production, and research on testicular biology in avians mainly focuses on chickens and ducks (Estermann et al., 2021). Research on circRNA in poultry mainly focuses on muscle cell proliferation and differentiation (Ouyang et al., 2018; Shen X. X. et al., 2019), follicle development (Shen M. M. et al., 2020; Wu et al., 2020), disease and immunity (Qiu et al., 2018; Wang et al., 2020) and intramuscular fat deposition (Wang, 2018). However, in the study of the genetic mechanisms regulating sperm motility, the mechanism of generation and the downstream functions of circRNAs remain unclear.

Studies have shown that in mammals, the brain and testis are the tissues with the highest expression levels of circRNAs(Zhou et al., 2018; Mahmoudi and Cairns, 2019). The normal progression of testicular development and spermatogenesis depends on the precise regulation of related genes at the transcriptional and post-transcriptional levels, and ncRNAs can be temporally and spatially refined. At present, research on sperm motility regulation, especially non-coding RNA-mediated gene regulation, is relatively scarce. In this study, based on the data on semen quality and egg fertilization rate, male Yili geese with extreme differences in sperm motility were strictly screened. High-throughput sequencing technology was used for the first time to study the circRNA expression profile of the testicular tissue of Yili geese with high and low sperm motility, and a total of 26,312 circRNAs were identified. Most of these circRNAs were 23 bp-1,511 bp in length, with an average length of 357 bp, which is similar to the length of circRNAs identified in other animals (Liang et al., 2017; Yuan et al., 2018). It is worth noting that in this study, circRNAs were relatively evenly distributed in intergenic, exonic and intronic regions, and most of them were derived from intergenic regions (34.19%). Tang et al, (2020) and other studies have shown that circRNAs are abundantly expressed in male germ cells during spermatogenesis, and circRNA levels increase with the progress of spermatogenesis. This study identified a higher number of circRNAs in high sperm motility testes, indicating that there are more sperm cells in testes with high sperm motility, that is, the spermatogenesis ability is stronger. In addition, compared with the low sperm motility group, a total of 173 DECs were screened, of which 82 were up-regulated and 91 were down-regulated. The source genes of these DECs are widely involved in biological processes such as growth and development, reproduction, and metabolism.

To explore the relationship between these DECs and sperm motility in Yili geese, GO enrichment analysis was performed on the host genes of these circRNAs, and it was found that they were mainly involved in biological processes (63.79%), and were significantly enriched in the reproduction of a single-celled organism, cytoskeletal protein binding, actin binding, adenyl nucleotide binding, microtubule organizing center and other terms. Signaling pathways that play an important role in sperm motility are included in the significantly enriched signaling pathways. Among them, actin is an important part of the cytoskeleton (Hohmann and Dehghani, 2019), and is distributed in mammalian cells, including Sertoli cells in the testis, and actin polymerization in the sperm tail during capacitation regulates motility (Finkelstein et al., 2013). The core of sperm flagella is made up of microtubules, along with tens of thousands of tiny molecular motors called dyneins, which bend these microtubules rhythmically, creating wave after wave of motion, resulting in steering. Sudarshan (Gadadhar et al., 2021) and others found that a special enzymatic modification of tubulin, called glycylation, is the key to keeping sperm moving in a straight line. It is suggested that these circRNAs may be involved in the regulation of sperm motility.

KEGG enrichment analysis showed that autophagy-animal and ubiquinone and other terpenoid-quinone biosynthesis were the most significantly enriched pathways (*p* < 0.05), and the source genes of differential circRNAs involved in these two pathways were *Pdpk1* (phosphomuscle alcohol-dependent protein kinase 1) and *COQ5* (methoxy-6-polyacryl-1,4-benzoquinone methylase). The *PDPK1* gene is expressed in spermatogenic cells, and PDPK1 is localized in the post-acrosomal region of mouse epididymal caudate sperm (Vadnais et al., 2013). *COQ5* is a methyltransferase with adenopicromethionine as a methyl donor. It is located on the stromal side of the inner mitochondrial membrane and plays a crucial role in the stability and activity of the CoQ protein complex (Nguyen et al., 2014). CoQ may not only participate in electron transfer in the mitochondrial respiratory chain of cells, maintaining the functions of cellular respiration and energy metabolism, but also participate in the redox reaction of the body as an antioxidant (Zhong and Zhong, 2014).

Autophagy is a basic process that exists in all eukaryotes, and is involved in the life processes of various cells in the male reproductive system, as well as being involved in the key pathophysiological processes of many diseases of the male reproductive system, such as azoospermia, oligospermia, asthenozoospermia, cryptorchidism, and orchitis (Zhu et al., 2019). Lei et al, (2021) studied the initiation of autophagy in mouse testis and found that after autophagy was blocked in sperm cells, the assembly of a series of key structures during sperm differentiation was disrupted. For example, the deformed scaffold of the sperm–sperm collar (manchette) and the tail shaft (axoneme) to ensure sperm movement at the same time lead to a large amount of cytoplasm to remain in the sperm head that ought to have been removed, resulting in morphological deformities and movement disorders. Yefimova et al, (2013)abolished autophagy by germ-cell-specific knockout of *Atg7*, resulting in reduced testicular weight, sperm deformities, and significantly reduced fertility in male mice. Liu et al, (2016) found that Sertoli cell-specific knockout of *Atg5* or *Atg7* disrupted autophagy, resulting in disorders of the vas deferens and deformed sperm heads, which in turn affected the reproductive performance of male mice.

Coenzyme Q (coenzyme Q, CoQ) is a class of quinone substances widely distributed in living organisms. Benzoquinone, also known as ubidearenone and ubiquinone, is a lipid-soluble antioxidant mainly present in the mitochondria, and is also a natural antioxidant and free radical scavenger in mammals (Lafuente et al., 2013; Liu et al., 2017). As an energy promoter and antioxidant, coenzyme Q10 is mainly distributed in the mitochondria in the midsection of sperm, and the availability of the energy required for sperm motility and other energy-dependent processes depends on the availability of coenzyme Q10 (Aby and Lavon, 1997). Studies have shown that sperm concentration, motility and semen parameters are related to *CoQ10* concentration, as CoQ10 can reduce stress oxidation, increase antioxidant enzyme activity, and improve overall antioxidant capacity (Balercia et al., 2009; Lafuente et al., 2013). Studies have confirmed that ubiquinone (Q10) coenzyme is more active in the biosynthesis of the male testis and female follicular fluid, and confirmed that coenzyme Q10 is inseparable from mammalian reproduction (Shetty et al., 2013; Varela-Lopez et al., 2017). Huo Min (Huo, 2018) added different concentrations of *CoQ10* exogenously to a frozen dilution solution, and detected various indicators of cashmere goat sperm after thawing. The results show that compared with the control group, the sperm quality after freezing and thawing was significantly improved (*p* < 0.05). When the concentration of coenzyme Q10 was 400 μg/ml, the sperm motility rate was 60.2%, which was significantly higher than that of the control group (*p* < 0.05), and the intracellular ROS content was significantly decreased (*p* < 0.05); when the concentration of coenzyme Q10 was 40 μg/ml, the sperm plasma membrane integrity rate was 63.0%, the acrosome integrity rate was 76.5%, and the mitochondrial membrane potential was 2.9, which were significantly higher than the levels found in the control group (*p* < 0.05). Hamed et al, (2019) explored the protective effect of coenzyme Q10 (*CoQ10*) and berberine (BB) combined with and without varicocelectomy on sperm parameters in rats with postoperative varicocele. They showed that BB and *CoQ10* alone and/or together could improve sperm parameters and reduce sperm DNA damage in varicocele-induced rats. It is worth noting that although the metabolic pathways in the KEGG enrichment were not significantly enriched, the pathways with the most enrichment of source genes were identified. These results indicate that there are cellular differences in testicular tissue in Yili geese with high and low sperm motility, and many of the DECs identified may play a roles in regulating autophagy, anti-oxidation and cell metabolism, thereby participating in the regulation of sperm motility in Yili geese.

The circRNA-miRNA-mRNA interaction network further revealed that cirRNA may regulate the expression of mRNA through the interaction with miRNA to regulate the sperm movement ofYili geese. In the interaction network, miR-212–5p, which has the most binding sites for circRNAs, was reported to be developmentally related to the follicles of ewe during FSH stimulation (Hya et al., 2022). Spermatogonial stem-cell-specific miR-221 is involved in the regulation of spermatogenesis in mice (Smorag et al., 2012). Qin et al, (2019) confirmed that circRNA-9119 is a regulatory circRNA involved in testicular inflammation and acts as a sponge for miR26a and miR-136 in Sertoli cells and Leydig cells in response to mimic RNAs, and that the stimulation of viral replication produces poly I:C. These results indicate that circRNAs have potential roles in the regulation of testicular development or sperm motility in Yili geese, and whether these circRNAs really affect testicular development or sperm motility requires further experiments in order to study and verify their mechanisms.

## 5 Conclusion

In summary, this study analyzed the expression profile of circRNAs in the testis of Yili geese with high and low sperm motility, identified a large number of circRNAs in the testis, revealed the genomic characteristics and length distribution of circRNAs, and constructed the circRNA-miRNA interaction network. The results suggest that circRNAs may regulate the sperm motility of Yili geese through interaction with miRNAs, which provides a solid foundation for identifying and characterizing the key circRNAs involved in testis development or the regulation of sperm motility.

## Data Availability

The data presented in the study are deposited in the BioProjectin NCBI repository (https://www.ncbi.nlm.nih.gov/bioproject/PRJNA856143), accession number is PRJNA856143.
