# Supplementary material for: Differential expression of circRNAs of testes with high and low sperm motility in Yili geese
Source: Front Genet. 2022 Sep 26;13:970097. doi: 10.3389/fgene.2022.970097 (PMC9548634; doi:10.3389/fgene.2022.970097)
Supplement: Supplementary file 2 [file Table1.DOCX]

TableS1. RT-PCR Primers of the differentially expressed circRNAs

| Gene | Primer Sequence | Product Length | Annealing Temperature |
| --- | --- | --- | --- |
| novel_circ_0042868 | F:CATGGTTGCTGCTGCTTTCTG | 144 | 60.53 |
|  | R:GCCATGCTCTGTAACGGGAA |  |  |
| novel_circ_0030568 | F:GACAAATGCTGCACTTCCCA | 106 | 59.71 |
|  | R:GGCGACACAGCGAGTTTTTC |  |  |
| novel_circ_0013769 | F:TGCCTGGATTCGCCATTCTT | 121 | 59.97 |
|  | R:AGCCGATCCGTAACGTCTTC |  |  |
| novel_circ_0007998 | F:TTTCCCCAGACTTTCACCTTGT | 188 | 60.03 |
|  | R:GCCCCACCATTTGTTGCAC |  |  |
| novel_circ_0017590 | F:TGCGAGATGAGCCCTTTTTG | 187 | 59.55 |
|  | R:TTTGCTGTTCCAGTCCTAGCC |  |  |
| novel_circ_0018059 | F:CTGCTTTAATGCCAGCCACAT | 112 | 59.45 |
|  | R:TTGACTTTCTCCTCCCAGCTC |  |  |
| novel_circ_0034225 | F:GTGGAGAATCAGTCACAGTGGAAA | 161 | 61.30 |
|  | R:GACTTCAGCACGCAGGGTTG |  |  |
| novel_circ_0037707 | F:GCTGGCTGAGTTACCTTTGTG | 117 | 59.46 |
|  | R:TCTACGTAAAGGGCCAGGTTC |  |  |
| GAPDH | F:TGATGCTCCCATGTTCGTGATG | 168 | 60.66 |
|  | R:TGATGCTCCCATGTTCGTGATG |  |  |

Note: F: for upstream primer R: for downstream primer
